# Therapeutic Potential of Targeting Complement C5a Receptors in Diabetic Kidney Disease

**DOI:** 10.3390/ijms24108758

**Published:** 2023-05-15

**Authors:** Inez A. Trambas, Melinda T. Coughlan, Sih Min Tan

**Affiliations:** Department of Diabetes, Central Clinical School, Monash University, Melbourne, VIC 3004, Australia

**Keywords:** complement system, diabetic kidney disease, inflammation

## Abstract

Diabetic kidney disease (DKD) affects 30–40% of patients with diabetes and is currently the leading cause of end-stage renal disease (ESRD). The activation of the complement cascade, a highly conserved element of the innate immune system, has been implicated in the pathogenesis of diabetes and its complications. The potent anaphylatoxin C5a is a critical effector of complement-mediated inflammation. Excessive activation of the C5a-signalling axis promotes a potent inflammatory environment and is associated with mitochondrial dysfunction, inflammasome activation, and the production of reactive oxygen species. Conventional renoprotective agents used in the treatment of diabetes do not target the complement system. Mounting preclinical evidence indicates that inhibition of the complement system may prove protective in DKD by reducing inflammation and fibrosis. Targeting the C5a-receptor signaling axis is of particular interest, as inhibition at this level attenuates inflammation while preserving the critical immunological defense functions of the complement system. In this review, the important role of the C5a/C5a-receptor axis in the pathogenesis of diabetes and kidney injuries will be discussed, and an overview of the status and mechanisms of action of current complement therapeutics in development will be provided.

## 1. Introduction

The estimated prevalence of diabetes mellitus has increased threefold over the past two decades, from approximately 150 million at the turn of the century to over 530 million today [1]. The two most common forms of diabetes mellitus are type 1 (T1DM) and type 2 (T2DM). T1DM is an autoimmune disease in which insulin-secreting pancreatic β-cells are targeted, leading to a loss of insulin secretion and subsequent impaired glucose metabolism, causing hyperglycemia [2]. The cause of T2DM is more complex, with genetic and lifestyle factors, among other variables, contributing to the development of impaired glucose metabolism and hyperglycemia [3]. Both individuals with T1DM and T2DM are at risk of developing diabetic complications [4,5], and between 30 and 40% will develop diabetic kidney disease (DKD) [6,7,8,9].

## 2. Diabetic Kidney Disease

The diabetes-induced chronic hyperglycemic milieu promotes alterations across metabolic, hemodynamic, and inflammatory pathways [10,11]. Hyperglycemia dysregulates blood pressure and vascular resistance due to renin-angiotensin-aldosterone system (RAAS) activation [12]. The resultant hypertension, a primary hallmark of DKD, is especially detrimental to the kidney [13]. The overproduction of pro-inflammatory mediators, including reactive oxygen species (ROS), advanced glycation end products (AGEs), cytokines, chemokines, and complement proteins, induces a positive feedback loop of chronic inflammation [4,14,15]. Transforming growth factor β (TGF-β), connective tissue growth factor (CTGF), and vascular endothelial growth factor (VEGF) promote collagen and fibronectin deposition and the replacement of the normal tissue architecture with extracellular matrix (ECM) proteins across the kidney [16,17]. These renal structural changes activate intracellular signaling pathways that promote the expansion of the mesangial matrix, glomerular hypertrophy, tubulointerstitial fibrosis, and glomerulosclerosis [10,18]. Microalbuminuria and worsening hypertension are the first clinical markers of disease development [19,20], and as the glomeruli fail to filter the blood, proteinuria becomes severe and persistent [5]. The estimated glomerular filtration rate (eGFR), a measure of kidney function, continues to decline as the disease progresses.

Unfortunately, current measures to treat DKD—reducing hypertension, managing cardiovascular disease, making dietary changes, and avoiding nephrotoxins—do not stop progression to ESRD. In the absence of robust biomarkers for early detection of those likely to develop DKD, the available treatments are employed after kidney function has already begun to decline. Novel biomarkers, in addition to therapeutic targets, are critical if diagnosis, intervention, and treatment are to be improved before renal damage is irreversible and to reduce the significant morbidity and mortality associated with DKD.

A large and accumulating body of evidence indicates that activation of the complement system is increased in preclinical models of diabetes and in clinical samples from individuals with diabetes and may be a biomarker for DKD development [21,22,23,24,25,26,27].

## 3. The Complement System

**Figure 1:**
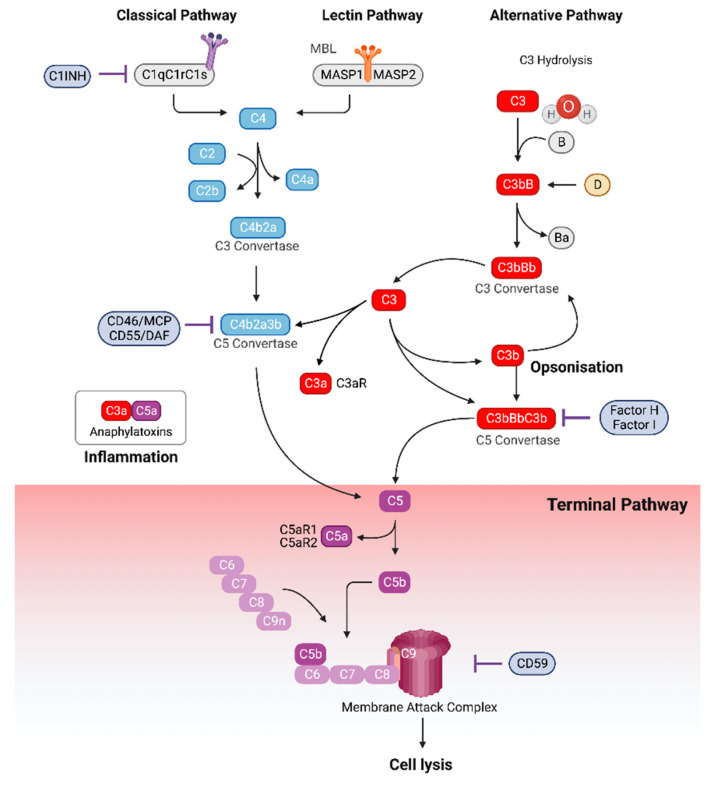
The complement cascade. Three pathways of complement activation, the classical, lectin, and alternative pathways, converge at the formation of C3, initiating the common terminal pathway. Cleavage of C3 leads to the formation of complement products C3a, an anaphylatoxin that binds the receptor C3aR, and C3b, an opsonin, in addition to contributing to the formation of C5. Cleavage of C5 leads to the formation of C5a, the most potent anaphylatoxin, which binds receptors C5aR1 and C5aR2, and C5b, which forms part of the membrane attack complex (MAC) through polymerization with complement components C6, C7, C8, and C9. The pore-forming MAC inserts itself into cell membranes, inducing osmotic damage and cell lysis. The complement cascade is regulated by a range of membrane-bound and soluble inhibitors, including C1INH, MCP, DAF, factor H, factor I, and CD59. Abbreviations: CRP, C-reactive protein; MCP, membrane co-factor protein; DAF, decay accelerating factor; MASP, MBL-associated serine protease.

The complement system is a highly conserved element of the innate immune system. Over 40 circulating proteins, surface-bound receptors, and plasma proteases comprise the complement cascade, which is activated by three distinct pathways, as described elsewhere [28,29,30,31]. As illustrated in Figure 1, the classical, lectin, and alternative pathways all converge at protein C3 and share a common downstream signaling cascade termed the terminal pathway. Cleavage of C3 generates the effector proteins C3a and C3b. C3a is an anaphylatoxin that induces inflammatory signaling upon binding its receptor, C3aR, expressed on various immune and non-immune cells [32,33]. C3b acts as an opsonin, supporting phagocytic cells to engulf their targets [33]. Further, the C3b fragment can also form part of the alternative pathway C3 convertase (C3bBb), creating an amplification loop of complement activation.

Downstream formation of the C5 converses (C4b2a3b and C3bBbC3b) and subsequent cleavage of C5 produce C5a and C5b. C5a is an anaphylatoxin 50-fold more potent than C3a [34], which binds its receptors C5aR1 and C5aR2. C5b associates with complement proteins C6, C7, C8, and C9, polymerizing to create the membrane attack complex (MAC), a pore-forming molecule that inserts itself into plasma membranes, triggering osmotic shock and cell lysis. Thus, the three results of complement activation are broadly inflammation through the anaphylatoxins C3a and C5a, opsonization through C3b and subsequent phagocytic action, and cell lysis mediated by the MAC.

The complex physiology and function of the kidney render it especially vulnerable to complement-mediated injury, and as a consequence, systemic complement dysregulation often results in isolated kidney disease [31]. Furthermore, a number of studies have highlighted that intrarenal synthesis and activation of complement proteins are crucial mediators in the pathogenesis of both acute [35,36] and chronic kidney injuries [37,38,39]. The increasing recognition of the complement system as a pathophysiologic mediator of renal injury emphasizes the importance of targeting complement for the treatment of various renal conditions.

## 4. The Hyperglycemic Milieu Promotes Complement Activation

The chronic hyperglycemic setting that characterizes diabetes is believed to promote complement activation. Post-translational protein modifications, in particular glycation, may prompt activation of the lectin pathway through association of MBL with the sugars that have been deposited on cells [40,41,42]. Elevated serum C5b-9, indicative of the MAC, is frequently reported in patients with diabetes [43,44]. Glycation of the complement inhibitor CD59 renders the glycoprotein unable to bind C9 and thus unable to inhibit the formation of the MAC, which may also contribute to excessive complement-mediated damage in diabetes [45]. AGEs have also been reported to bind C1q, activating the classical pathway [46]. While the precise molecular mechanisms underpinning complement activation in diabetes and its complications remain to be fully elucidated, a significant body of evidence indicates complement proteins are dysregulated in patients with diabetes and its complications, as summarized in Table 1. Increased levels of circulating MBL are associated with the development and progression of albuminuria in patients with both T1DM and T2DM [47,48,49,50,51,52,53], while increased levels of anaphylatoxins C3a and C5a are associated with renal function decline [43,44]. Moreover, deposition of complement proteins C1q, C3, C3c, and C4d in the kidney is associated with DKD, worsening albuminuria, and the progression to ESRD [26,46,54,55]. Further, urinary complement C3 and C9 are negatively correlated with eGFR and associated with progressive renal decline in patients with diabetes [54,56].

Given the complexity of the complement cascade, comprised of inflammatory mediators and their receptors, regulatory proteins, and intrinsic factors, the measurement of individual complement proteins often does not adequately reflect the biology and activity of the complement system in its entirety. As such, a reproducible systematic complement system assaying protocol capable of measuring total complement activity in addition to individual complement components needs to be established in order to fully investigate this intricate system [57]. Such a protocol would aid in both the development of complement therapeutics as well as improve laboratory techniques to investigate complement as a biomarker of disease progression.

**Table 1:** Evidence of complement activation in patients with diabetes and DKD.

References	Biospecimen Type	Complement Protein	Key Finding/s
[46,54]	Renal tissue	C4d	C4d deposition associated with diabetic nephropathy and correlated with disease severity
[46,54]	Renal tissue	C1q	C1q deposition associated with renal dysfunction and disease progression
[46]	Renal tissue	C5b-9	MAC deposits associated with diabetes and correlated with severity of disease
[21,26,55]	Renal tissue	C3	Increased C3 deposition or expression associated with kidney function decline (including albuminuria) and/or progression to ESRD
[43]	Renal tissue	C5	C5 deposition increased in diabetes vs. non-diabetic controls
[43,44]	Plasma	C3a	Plasma C3a is significantly elevated in diabetic disease and DKD, and is associated with albuminuria
[43,44]	Plasma	C5a	Plasma C5a is significantly elevated in the diabetic milieu and is associated with reduced eGFR
[43]	Plasma	sC5b-9	Circulating C5b-9 is upregulated in DKD versus diabetic patients without kidney involvement
[47,48,49,50,51,52,58]	Serum	MBL	MBL levels are associated with the progression and/or progression of albuminuria in patients with T1DM and T2DM
[53]	Serum	MAp19	MAp19 concentration associated with increased risk of progression of albuminuria in T1DM patients
[59]	Serum	C7	Serum C7 levels are increased in patients with early diabetic nephropathy in comparison to controls
[54,56]	Urine	C3	Urinary C3 abundance is negatively correlated with progressive decline in eGFR; urinary C3 is elevated in DKD versus diabetes alone
[54]	Urine	C9	Urinary C9 abundance is negatively correlated with progressive decline in eGFR
[56]	Urine	C3b	Urinary C3b significantly increased in DKD versus diabetes alone

Currently, it is not clear which activation pathway contributes predominantly to complement activation in diabetes. Bus and colleagues investigated complement expression in autopsied renal biospecimens from individuals with T1DM or T2DM, in addition to non-diabetic controls [46]. Renal biospecimens from patients with diabetes had increased C4d deposits, which correlated with reduced eGFR. Further, C1q was observed in over one-third of the diabetic samples and almost absent in non-diabetic controls, indicating activation of the classical pathway [46]. Additionally, patients with DKD also had increased kidney IgM deposition, further strengthening evidence that the classical pathway is likely activated in diabetes. Analyses of gene expression reveal that complement proteins C3, C1q, MBL, and C5b-9 are increased in patients with DKD in comparison to non-diabetic controls [60]. Increased deposition of complement C3 and C1q has Increased kidney function decline has been reported in patients with C3 and C1q deposits [60], and C3c deposition has been identified as an independent risk factor for the progression to ESRD [55]. Moreover, in vitro studies indicate the proximal tubules are able to activate the alternative pathway via intrinsic convertase activity [61], and a lack of complement regulator expression may exacerbate complement-mediated local tissue damage [62]. Nevertheless, each activation pathway converges on the shared terminal pathway, making effectors of the terminal pathway ideal therapeutic targets for the prevention of complement-mediated injury.

## 5. C5a and C5a Receptors

C5a is the most potent anaphylatoxin produced by the complement system. It induces diverse immunological effects through binding its receptors, C5aR1 and C5aR2. With functions across chemotaxis, adhesion, migration, and cell arrest, C5a also modulates cytokine profiles and promotes the production of ROS [63,64]. Despite human C5aR2 sharing 58% amino acid sequence homology with C5aR1 [65], differences in structural regions important for G protein coupling and receptor internalization [66,67,68,69] render the receptors distinct from one another [70]. The key differences between C5aR1 and C5aR2 in terms of signaling, expression, and localization are summarized in Table 2.

## 6. C5a-C5aR1 Signaling

**Figure 2:**
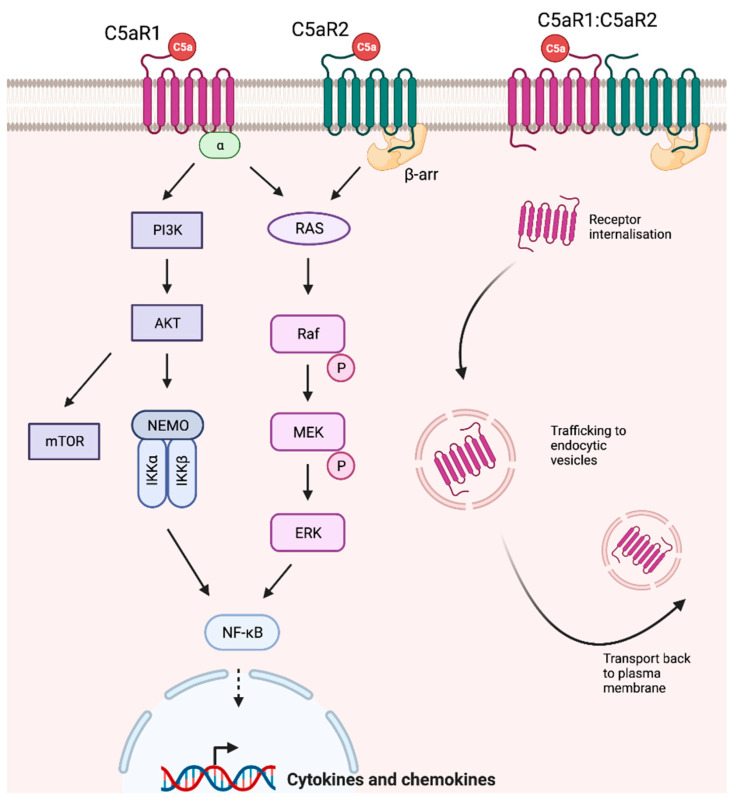
C5a receptor signaling. C5aR1 is a canonical G protein-coupled receptor (GPCR) that predominantly mediates signal transduction through Gα family proteins. Downstream signaling through PI3K or Ras MAPK pathways regulates cell growth, motility, metabolism, survival, and cytokine production. C5aR2 preferentially signals through β-arrestins, activating the downstream transcription factor NF-κΒ. C5aR1 and C5aR2 may also form heteromers and recruit β-arrestins; however, the precise signaling pathways and intracellular trafficking pathways are currently poorly characterized.

C5aR1 (CD88) is a canonical seven-transmembrane (7TM) G protein-coupled receptor (GPCR) predominantly expressed on the cell surface [69,71]. Activation of the receptor induces downstream G protein-mediated ERK 1/2 phosphorylation in addition to β-arrestin signaling pathways [43,72], as illustrated in Figure 2. C5aR1 is expressed across cells of the myeloid lineage [73], in addition to epithelial and endothelial cells and subsets of T and B lymphocytes [68]. In the healthy kidney, C5aR1 protein is expressed in tubular epithelial cells [74,75], with strong expression in the basolateral distal tubule and weaker expression in the proximal tubules [76]. Renal mRNA expression has also been reported in glomerular cells and vascular endothelial cells [74], though protein expression in podocytes is scarce [77].

## 7. C5aR1 Promotes Inflammation and Tissue Damage in Diverse Models of Acute Kidney Injury

In the diseased or injured kidney, C5aR1 expression is significantly altered [74,75,76]. In biopsied renal biospecimens from transplant patients with delayed graft function, staining revealed a diffuse pattern of C5aR1 expression in the proximal and distal tubules [75]. In contrast, minimal or focally positive staining in the distal tubules was identified in control biospecimens. In preclinical models, the induction of murine ischemic injury is associated with an increased expression of C5aR1 in the tubular cells as well as in infiltrating neutrophils, while in control mice, C5aR1 is predominantly expressed in the mesangial cells [78]. A number of preclinical studies indicate that genetic ablation or pharmacological inhibition of C5aR1 decreases markers of renal injury, immune cell infiltration, and fibrosis in renal IRI [29,75,79,80], unilateral uretal obstruction (UUO) models of obstructive nephropathy and fibrosis, uropathogenic *Escherichia coli* (UPEC)-induced pyelonephritis [81,82], and folic acid (FA)-induced AKI [73]. These studies highlight the pathogenic role the receptor plays in acute renal injury.

**Figure 3:**
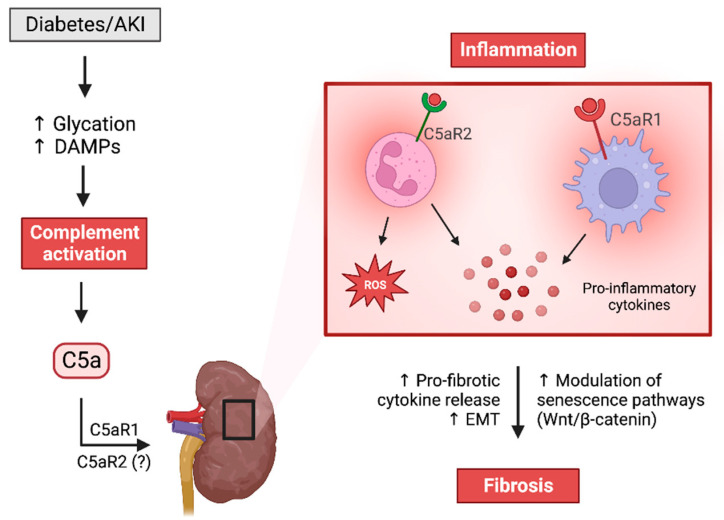
C5a receptors in renal inflammation and fibrosis. Complement activation and downstream production of the potent anaphylatoxin C5a promote the recruitment of inflammatory cells to the renal compartments. Local production of pro-inflammatory cytokines and chemokines, such as MCP-1 and IFNγ, exacerbates inflammation and prompts the release of pro-fibrotic cytokines and mediators, such as TGF-β and CTGF. These mediators promote fibrosis through collagen and fibronectin deposition and the replacement of the normal renal architecture with extracellular matrix proteins. Renal function declines as fibrosis becomes more severe.

A proposed mechanism of C5a-C5aR1/2-induced renal injury is summarized in Figure 3. Complementary activation and production of the powerful chemoattractant C5a recruit inflammatory cells, particularly macrophages and neutrophils, to the site of injury. Renal C5a may modulate macrophage polarization to an M1 phenotype via C5aR1, promoting the production of pro-inflammatory cytokines and ensuing inflammation and fibrosis [83]. The C5a/C5aR1 signaling axis has also been reported to stimulate the activation and proliferation of renal fibroblasts, in addition to upregulating gene expression of pro-fibrotic mediators PDGF and TGF-β in renal tubular epithelial cells [79]. These mediators promote EMT, contributing to fibrosis [79]. Interestingly, C5a has been shown to stimulate ROS production via NOX-dependent pathways in mouse kidney endothelial cells [84], and further, C5a stimulation can induce pyroptosis in renal podocytes [85]. Collectively, these mechanisms promote the activation and proliferation of renal and immune cells, exacerbating inflammation and fibrosis, which ultimately leads to renal injury and kidney dysfunction.

## 8. C5aR1 Mediates Pathogenesis in Diabetic Kidney Disease

C5aR1 plays a pathogenic role in diverse preclinical models of both T1DM and T2DM. In *db*/*db* mice, a murine model in which mutations in Lepr^db^ render the mice diabetic, modeling a T2DM-like phenotype, C5 and C5aR1 expression are upregulated in comparison to non-diabetic *db*/*m* mice [44,86]. Furthermore, treatment with C5a or C5aR1 antagonists has been reported to significantly attenuate renal injury [44,86]. Yiu and colleagues observed that blockade of C5a with a novel mixed RNA/DNA a-tamer, NOX-D21, markedly reduced fibrotic injury, including tubulointerstitial fibrosis and glomerulosclerosis [87]. Of note, NOX-D21 did not have any observable impact on proteinuria. The predominantly tubular-specific expression of C5aR1 and lack of expression in the glomeruli, as the group proposed, may account for the absence of an effect on proteinuria observed [87]. Interestingly, a number of other studies using C5aR1 antagonists in *db*/*db* mice have reported that the interruption of the C5a-C5aR1 signaling axis reduces albuminuria.

The administration of the C5aR1 antagonist W-54011, a potent and orally active non-peptide antagonist, to *db*/*db* mice significantly attenuated renal injury, with reductions in pro-inflammatory cytokines IL-6 and MCP-1 decreases in mRNA expression of pro-inflammatory gene markers TLR2, MCP-1, and macrophage marker F4/80 in *db*/*db* kidneys [86]. These changes were independent of glucose and lipid metabolism pathways. Similarly, previous studies from our laboratory underscore the protective benefit achieved by disrupting the C5a/C5aR1 signaling axis in murine models of diabetes [44]. Mice modeling streptozotocin-induced type 1 diabetes treated with the C5aR1 antagonist PMX53, an orally active peptide, experienced significant reductions in inflammation, oxidative stress, and tissue damage, as indicated by reductions in albuminuria, urinary 8-isoprostane, and cytokines such as IL-18 [44]. Further, PMX53 administration also reduced macrophage infiltration into the tubulointerstitium of diabetic mice.

Emerging research suggests C5a may play a role as an epigenetic mediator capable of modulating the expression of genes involved in cellular senescence pathways [88,89]. C5a stimulation in renal tubular epithelial cells has been found to upregulate gene expression of senescence mediators, particularly mediators from the Wnt/β-catenin pathway [88], which is associated with the progression of AKI to chronic kidney disease, including DKD [90,91]. Moreover, inhibition of C5aR1 with PMX53 attenuates cortical expression of genes involved in cellular senescence, in addition to reducing markers of the inflammatory senescence-associated secretory phenotype (SASP) in streptozotocin-induced diabetic mice [89]. Thus, in addition to the direct effects of C5a/C5aR1 signaling in inflammatory cells in terms of chemotaxis and the release of chemokines and cytokines, this axis may also exacerbate renal inflammation and injury through modulating cellular senescence.

These important preclinical findings highlight the potential of targeting the anaphylatoxin receptor signaling pathway in the treatment of DKD. Unlike anti-C5 inhibitors, C5a and C5aR1 antagonists preserve the upstream functions of C3a and C3b as well as the downstream formation of the MAC, a critical defense against microbial infection. Thus, targeting complement at this axis may balance the attenuation of excessive inflammation with the preservation of immune defense functions. However, the potential contribution of the second receptor for C5a, C5aR2, must be elucidated before the therapeutic potential of inhibiting the C5a-signaling axis may be realized.

## 9. C5a-C5aR2 Signaling

Until Ohno et al. discovered C5aR2 in 2000 following the cloning of the *c5ar2* gene into a human immature dendritic cell line [92], C5aR1 was believed to be the only receptor for C5a. The then orphan receptor was subsequently characterized with genetic sequencing and transfection studies and found to share significant homology with C5aR1 and other GPCRs, which inspired the name C5L2—“C5a-like receptor 2” [92]. Over the past two decades, the receptor has been labeled a scavenging, non-signaling decoy receptor [67,93], an anti-inflammatory receptor [71,94,95,96,97,98], and more recently a pro-inflammatory receptor [99,100,101,102,103,104]. The great variety in experimental designs, in addition to the lack of selective C5aR2 ligands, both agonists and antagonists, has led to great discrepancies in the findings of early C5aR2 research. Today, the signaling pathways and molecular functions of C5aR2 remain poorly understood.

C5aR2 is a 7TM GPCR that binds both complement proteins C5a and C5adesArg with high affinity [70,105,106]. Sharing 37% homology with GPCR C5aR1, C5aR2 is unusual in that it has not been found to couple G proteins [70,105,107]. Initially, the absence of observed G protein-coupling was believed to be indicative of a lack of downstream signaling. Recent advances in GPCR research have revealed that the signaling pathways of these receptors are far more complex than once thought. GPCRs may engage various G proteins or signal independently from them [108,109], as seems to be the case with C5aR2. In fact, C5aR2 is one of many unusual GPCRs that preferentially signal through alternative scaffold proteins [110,111]. C5aR2 recruits β-arrestins [32,112,113,114], and modulates downstream ERK1/2 signaling pathways across various cell types, including human monocyte-derived macrophages (HMDMs) [113], neutrophils [115], and macrophages [116]. Further, C5aR1-C5aR2 heteromer formation can be induced by C5a stimulation, resulting in the recruitment of β-arrestins and ERK1/2 signaling [113,117,118,119]. In this heteromer formation, C5aR2 mediates C5aR1 internalization [114], though the specific trafficking pathways are currently unknown. In the kidney, C5aR2 is expressed in the renal tubules [76] and in resident inflammatory cells [120], though expression in mesangial cells has not been reported.

## 10. C5aR2 Promotes Inflammation in AKI

The role of C5aR2 in chronic kidney diseases, including DKD, still remains to be elucidated; however, a number of groups have reported the receptor contributes to inflammation in AKI.

Consistent with systemic complement activation following the induction of hypoxic injury [78,79,80,121,122,123], C5aR2 is upregulated in response to hypoxic conditions in vitro [75]. In order to investigate the potential contribution of C5aR2 to C5a-mediated damage in renal IRI, Poppelaars et al. generated both C5aR1^−/−^ and C5aR2^−/−^ mice [102]. Genetic ablation of C5aR2 was associated with significantly reduced levels of the inflammatory cytokines IL-6, TNF-α, and IL-1β, as well as the chemokines IL-8 and MCP-1. Moreover, knockout was protective against renal injury in a model of renal IRI, with knockout mice experiencing decreased acute tubular necrosis as well as reduced blood urea nitrogen levels, a marker of kidney dysfunction [102]. Interestingly, C5aR2 knockout was more protective against injury than C5aR1 knockout [102]. Thorenz et al. subsequently confirmed this important finding by modeling renal IRI in C5aR1^−/−^ and C5aR2^−/−^ mice [103]. While both knockout mice were protected against IRI-induced inflammation and demonstrated improved renal perfusion in comparison to wild-type mice, C5aR2^−/−^ mice showed significantly attenuated inflammation and fibrosis in comparison to C5aR1^−/−^ mice [103].

In a murine model of UPEC-induced acute pyelonephritis, C5aR2^−/−^ mice were protected against renal damage, as indicated by decreased renal KIM-1 and pro-inflammatory cytokines IL-1β and TNFα [120]. Abolition of C5aR2 led to significant reductions in intrarenal HMGB1 levels, a marker of cellular damage, reduced bacterial load in the kidney, and attenuated tubular damage in comparison to wild-type littermates [120]. Moreover, in macrophages from C5aR2^−/−^ mice lacking C5aR1 but expressing C5aR2, C5a stimulation led to increased levels of HMGB1 and inflammasome-related proteins NLPR3, ASC, and cleaved caspase-1, in conjunction with increased release cytokines of IL-1β and TNF-α [120]. These results indicate a role for C5aR2 in the expression and release of HMGB1, as well as activation of the multi-protein NLRP3 inflammasome in macrophages, which may contribute to renal inflammation independent of C5aR1 signaling.

At present, C5aR2 has never been studied in DKD. The few papers available in the current literature suggest a pro-inflammatory role for the receptor in AKI. It is important to note that individuals with diabetes are especially susceptible to AKI, and accumulating evidence suggests that AKI can progress to chronic kidney disease and DKD, as reviewed elsewhere [124,125]. Thus, since C5aR2 mediates the pathogenesis of AKI, it may also promote the progression of acute injury to chronic disease, though this remains to be fully elucidated.

## 11. Dysregulated C5aR2 Is Associated with Impaired Immunometabolism

Interestingly, a number of human studies have indicated that C5aR2 may contribute to dysregulated metabolism and chronic inflammation. These findings may provide an insight into the receptor’s potential role in DKD. A genome-wide association study identified the 19q13 region—the location of the *c5ar2* gene—as a putative risk area for the development of immunometabolic disorders by Marcil and colleagues in 2006 [126]. Subsequently, a single nucleotide polymorphism (SNP) S3231 in *c5ar2* has been reported in individuals with familial combined hyperlipemia, a disease characterized by increased cholesterol and triglycerides in the blood and insulin resistance [126]. The same SNP was later found to be associated with the loss of C5aR2 internalization, trafficking, and recycling [127]. A separate SNP (C698T) in *c5ar2* has been associated with the development of both coronary artery disease and T2DM in Chinese Han as well as Saudi populations [128,129,130,131]. The fact that *c5ar2* SNPs are associated with metabolic dysregulation, including cardiovascular disease and diabetes, may indicate a role for the receptor in diabetic complications, including DKD. C5aR2 has also been reported to be strongly expressed on human plaques in the chronic inflammatory condition atherosclerosis [132], a common comorbidity of diabetes. C5aR2 was predominantly expressed on infiltrating macrophages and positively correlated with plaque severity, suggesting the receptor promotes the chronic inflammatory milieu through modulating macrophage chemotaxis and cell arrest [132].

## 12. Therapeutic Targeting of C5 and the C5a-Signalling Axis

### 12.1. Eculizumab and C5-Targeted Therapeutics

Genome-wide association studies (GWAS) at the turn of the 21st century identified polymorphisms in the gene encoding complement factor H, a regulator of the alternative pathway, as a major risk factor in age-related macular degeneration [133,134]. This finding prompted intense interest in the potential contribution of complement to diseases of the vasculature. While the structure and function of the eye share few similarities with those of the kidney, the intricacy of the microvasculature predisposes both organs to complement-mediated attack [135].

The emergence of the anti-C5 monoclonal antibody Eculizumab marked the development of the first complement-targeted drug approved for clinical use [136,137]. Initially approved by the Food and Drug Administration in 2007 for the treatment of the rare blood disorder paroxysmal nocturnal hemoglobinuria (PNH) [135,138], and subsequently approved in 2011 to treat the rare kidney disease atypical hemolytic uremia (aHUS) [138], Eculizumab has been life-changing for patients. The great success of Eculizumab has prompted further trials in other diseases, including C3 glomerulopathy [139,140], IgA nephropathy [141], and renal transplant [142,143,144]. Antagonists directed against other complement proteins, including serine proteases and GPCRs, are in development.

Given the kidney’s unique susceptibility to complement attack, considerable research is being undertaken to elucidate the potential efficacy of complement inhibitors in the setting of various kidney pathologies, including renal transplant [145,146,147,148], lupus nephritis [149,150], IgA nephropathy [141,151], immune complex-mediated membranoproliferative glomerulonephritis [152,153], and C3 glomerulopathies [139,140,154,155]. The potential therapeutic benefit of targeting complement in chronic kidney pathologies, such as DKD, remains to be further explored.

Characteristic of all treatments that disrupt the immune system, targeting complement requires a careful balance between suppression of pathogenic activity and preservation of immune defense to be achieved [156]. Administration of Eculizumab, for example, is accompanied by an increased risk of meningococcal infection due to the downstream function of the MAC being abolished [157,158]. Prophylactic antibiotic use in conjunction with vaccination has reduced the incidence of infection in patients treated with Eculizumab as well as the anti-C5 monoclonals Ravulizumab [159,160] and Crovalimab [161]. The long-term impact of prolonged antibiotic use in these patients, however, requires further investigation. Furthermore, the necessity of high doses (Eculizumab 1200 mg) and biweekly intravenous administration [135] contributes to the treatment burden for patients, and the high cost of Eculizumab, approximately US$500,000 annually per patient, significantly restricts availability. The drawbacks associated with targeting C5 have prompted great interest in designing alternative complement inhibitors that do not promote an immunocompromised state and do not rely on intravenous administration.

### 12.2. Inhibition of C5aRs in Preclinical Models

**Figure 4:**
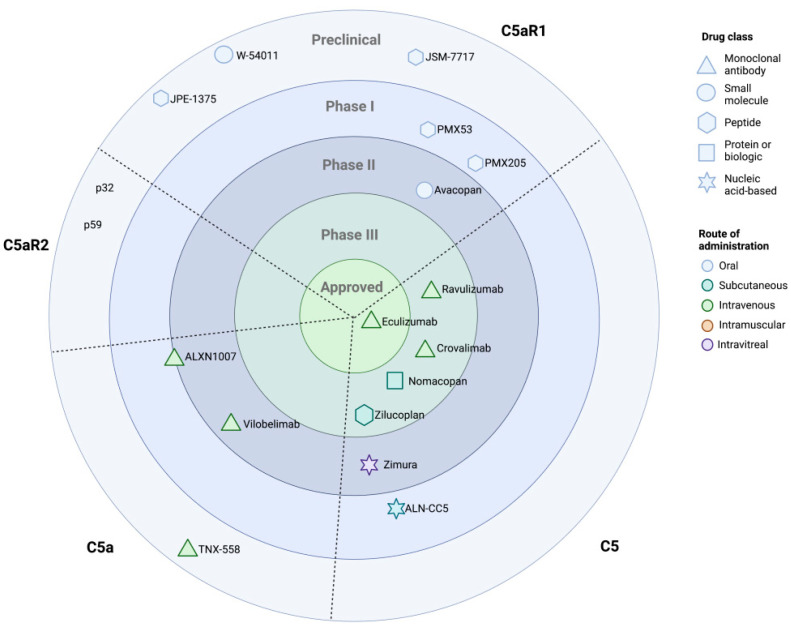
Complement inhibitors targeting the terminal pathway in development. The great success of the anti-C5 monoclonal antibody Eculizumab has prompted a renaissance in the development of complement therapeutics. In addition to monoclonals, a number of small molecules, peptides, biologics, and nucleic-acid-based aptamer drugs are in development for varied clinical applications. Despite the advances in developing drugs against C5, C5a, and C5aR1, there has been great difficulty in producing selective C5aR2 antagonists.

A number of orally active compounds against C5aR1, both peptides and small molecule drugs, are in varying stages of development, as outlined in Figure 4. Targeting at the C5a-signaling axis may rectify the adverse consequences of targeting C5 directly, as inhibition of C5a or its receptors preserves the downstream MAC in addition to the upstream actions of C3a and C3b. PMX53, an orally active peptide antagonist of C5aR1, has been trialed for safety in human studies [162], and has previously been tested in clinical trials in the context of rheumatoid arthritis [163]. Our laboratory has previously highlighted the protective benefit of inhibiting the C5a/C5aR1 signaling axis in a murine model of T1DM using PMX53 (Ac-Phe-[Orn-Pro-cha-Trp-Arg]) [44]. Administration of this orally active peptide significantly reduced albuminuria and oxidative stress in diabetic mice and decreased the severity of renal structural injury, including both glomerulosclerosis and mesangial matrix expansion [44]. PMX205 (hydrocinnamate-[Orn-Pro-dCha-Trp-Arg]) is a lipophilic analogue of PMX53 that demonstrates enhanced efficacy and stability in vivo in comparison to its parent molecule PMX53 [164,165], making the drug an ideal candidate for human disease. Both PMX53 and PMX205 have completed Phase I clinical trials, demonstrating safety in healthy volunteers [166,167]. While peptide-based drugs are generally associated with poor oral bioavailability and a short half-life in vivo due to enzymatic degradation, Xu and colleagues recently developed a technique to overcome these limitations and improve the function of PMX205 in vivo [168]. Through entrapping PMX205 within a biodegradable polymer-based nanoparticle, the group was able to increase the half-life twenty-fold in vivo in mice. Encapsulation in the nanoparticle did not interfere with the ability of PMX205 to inhibit C5a-induced ERK1/2 activity, and its functional activity was comparable to that of free PMX205 [168]. The establishment of this methodology promises to propel PMX compounds from early-phase clinical trials into the clinic. Moreover, such a technique may be used to improve the bioavailability of other peptide-based therapeutics.

JPE-1375 (Hoo-Phe-Orn-Pro-hle-Pff-Phe-NH2), a PMX53 derivative created through a hydrophobic substitution at the C-terminus of PMX53, is another well-characterized C5aR1 antagonist [169]. In a preclinical model of UUO-induced renal tubulointerstitial fibrosis, inhibition of C5aR1 with JPE-1375 was associated with a marked reduction in mRNA expression of pro-fibrotic mediators such as PDGF and TGF-β in the kidneys, as well as reduced collagen deposition [170]. These findings emphasize the significant benefit achieved through inhibiting C5a-signaling to reduce the development of fibrosis, a hallmark of DKD.

The promising results observed with peptide-based C5aR1 inhibitors in preclinical disease models, in addition to the safety and tolerability of PMX53 and PMX205 validated in Phase I trials, highlight the potential to advance these inhibitors into clinical practice for the treatment of various inflammatory conditions, perhaps including DKD. Moreover, the clinical approval and use of peptide-based drugs such as semaglutide, a glucagon-like peptide (GLP) analog for the treatment of T2DM, has generated major advances in large-scale peptide manufacturing to meet global demands [171]. As the limitations that once hampered the clinical translatability of peptide-based therapeutics continue to be overcome, the promise of these selective, stable, and low molecular weight compounds can be fully realized.

Results have recently been published for the open-label pilot trial investigating the orally active small molecule avacopan in the treatment of IgA nephropathy (IgAN), an antibody-mediated chronic renal condition associated with glomerulonephritis [172]. The majority of patients in the small cohort had reduced proteinuria following avacopan treatment, in addition to reduced MCP-1, a marker of inflammation. Moreover, avacopan was well tolerated and safe. While a randomized controlled study is required to assess avacopan use long-term, these promising results further underscore the clinical benefit of targeting C5a-signaling through C5aR1 inhibition in chronic kidney conditions such as DKD.

The contribution of C5aR2 to kidney disease requires further investigation, and is complicated by the controversies surrounding the receptor, including its ligands, cellular localization, signaling, and function, as reviewed elsewhere [70]. Furthermore, the lack of robust C5aR2-specific ligands, both agonists and antagonists, is a major barrier to investigating its potential therapeutic benefit in attenuating C5a-mediated inflammation. Fortunately, advances are being made in identifying C5aR2-specific ligands to aid investigation into the receptor’s function in health and disease. Two functionally selective C5aR2 peptide-based ligands, the partial agonists P32 and P59, were identified by Croker and colleagues [173]. Given the potential that C5aR2 mediates an anti-inflammatory role in some disease states, administration of a C5aR2 agonist may reduce C5a-mediated inflammation, though this requires validation in vivo. It is critical that the elusive role of C5aR2 in chronic renal conditions, including DKD, be fully elucidated in order to ascertain if there is a therapeutic benefit to targeting both C5a receptors in chronic inflammatory conditions.

## 13. Conclusions

Ten percent of global healthcare expenditure is spent on addressing the complications of diabetes [14], and today, DKD is the primary cause of end-stage renal disease (ESRD) [4,10]. The rapidly increasing prevalence of diabetes and the concomitant increase in diabetic complications such as DKD place diabetes at the forefront of 21st century challenges in healthcare. The unmet therapeutic need associated with conventional treatments highlights the urgency of novel biomarkers for enhanced diagnosis and early intervention, in addition to new therapeutic targets. Promising preclinical evidence indicates that targeting the complement system may be protective in DKD; however, a rigorous characterization of how complement expression and function contribute to disease pathogenesis is necessary to fully realize the potential therapeutic benefit of complement inhibition.

## Figures and Tables

**Table 2:** Comparison of the C5a anaphylatoxin receptors.

	C5aR1	C5aR2
Topology	Seven transmembrane receptor	Seven transmembrane receptor
G protein coupling	Coupling to G proteins of Gα family	No known coupling to Gα family proteins
β-arrestin recruitment	Recruitment of β-arrestins 1 and 2	Recruitment of β-arrestins 1 and 2
Expression	Immune cells: myeloid lineage, some lymphocytes Non-immune cells: epithelial cells	Immune cells of myeloid lineage with particularly strong expression on granulocytes Non-immune cells: neurons, fibroblasts, adipose cells, hepatocytes
Cellular localisation	Predominantly on cellular surface	Predominantly intracellular

## Data Availability

Data sharing is not applicable.
